# Qualitative study: burden of menopause-associated vasomotor symptoms (VMS) and validation of PROMIS Sleep Disturbance and Sleep-Related Impairment measures for assessment of VMS impact on sleep

**DOI:** 10.1186/s41687-021-00289-y

**Published:** 2021-04-26

**Authors:** Marci English, Boyka Stoykova, Christina Slota, Lynda Doward, Emad Siddiqui, Rebecca Crawford, Dana DiBenedetti

**Affiliations:** 1 Astellas Pharma Inc., Pharma Global Development, 1 Astellas Way, Northbrook, IL 60062-6111 USA; 2 Astellas Pharma Inc., Surrey, Chertsey UK; 3 RTI Health Solutions, Patient-Centered Outcomes Assessment Group, Research Triangle Park, NC USA; 4 RTI Health Solutions, Manchester, UK

**Keywords:** Menopause, Vasomotor symptoms, Sleep, Quality of life, PRO, Content validity

## Abstract

**Purpose:**

We evaluated the impact of menopause-associated vasomotor symptoms (VMS) on sleep. We also sought to establish the content validity of Patient-Reported Outcomes Measurement Information System (PROMIS) short form Sleep-Related Impairment and Sleep Disturbance measures in postmenopausal women with moderate to severe VMS.

**Methods:**

Cross-sectional, in-person, qualitative interviews were conducted in the United States (Texas, Illinois) and European Union (UK, France) with women aged 40–64 years experiencing moderate to severe VMS (≥35/wk). Main outcomes were impact of VMS on sleep based on concept elicitation and content validity of PROMIS Sleep-Related Impairment and Sleep Disturbance short forms via cognitive debriefing.

**Results:**

Thirty-two women (US: *n* = 16; EU: n = 16) participated. A majority (US: 93.8%; EU: 93.8%) said VMS affected sleep; specifically, they had sleep interrupted by sweating or overheating and had difficulty returning to sleep. Sleep disturbance was the most bothersome aspect of VMS (US: 75%; EU: 50%). VMS-associated sleep disturbance affected next-day work productivity, mood, relationships, daily activities, concentration, social activities, and physical health. Participants found both PROMIS sleep measures relevant and easy to answer; the Sleep Disturbance measure was considered the most relevant. Participants had no difficulty remembering their experiences over the 7-day recall period and found the response options to be distinct.

**Conclusion:**

VMS associated with menopause significantly interferes with sleep and next-day functioning (e.g., work productivity), supporting assessment of sleep outcomes in studies evaluating treatment of VMS. Women with moderate to severe VMS found that the PROMIS Sleep-Related Impairment and Sleep Disturbance short forms assessed constructs important to understanding sleep in the context of menopause-associated VMS.

**Supplementary Information:**

The online version contains supplementary material available at 10.1186/s41687-021-00289-y.

## Plain English summary

Vasomotor symptoms (VMS) associated with menopause have consistently been linked with poor sleep. This study confirms that sleep disturbance was the most bothersome aspect of VMS in women with moderate to severe VMS and negatively affected next-day work productivity, as well as women’s moods, relationships, daily activities, concentration, social activities, and overall physical health. The Patient-Reported Outcomes Measurement Information System (PROMIS) measure of sleep quality has been validated in the general population and in people with sleep issues, but not specifically in women with VMS associated with menopause. We found that the PROMIS Sleep-Related Impairment Short Form 8a (SRI SF 8a), which asks questions related to sleep-related impairment, and the PROMIS Sleep Disturbance Short Form 8b (SD SF 8b), which asks questions related to sleep disturbance, are relevant patient-reported outcome measures in women with VMS associated with menopause. Our findings confirmed the content validity of these scales in measuring the impacts of menopause-associated VMS symptoms in future clinical trials.

## Introduction

Vasomotor symptoms (VMS) associated with menopause (also known as hot flashes, hot flushes, and night sweats) can negatively affect quality of life and cause nocturnal awakenings that result in poor sleep [1, 2]. Self-reported rates of sleep difficulties range from 40% to 56% among perimenopausal or postmenopausal women, and the occurrence of VMS has consistently been linked with poor self-reported sleep, ranging from occasional to chronic and severe sleep difficulties [2]. Other VMS-related quality-of-life impairments include mood swings, poor concentration, reduced work productivity, disrupted relationships, social embarrassment, anxiety, and fatigue [1].

Previously published research indicates that treatments for menopause-associated VMS improve sleep outcomes [3–10]; however, there is a need for standardized, validated sleep questionnaires to evaluate sleep outcomes in future trials of VMS therapies. For example, a meta-analysis of seven placebo-controlled trials concluded that menopausal hormone therapy results in moderately improved sleep quality in women with VMS (standardized mean difference vs placebo: − 0.54; 95% CI: − 0.91 to − 0.18; *P* < 0.007) [3]. These studies used six sleep assessment tools, resulting in a range of sleep outcomes. The authors concluded that standardized scales should be used to assess self-reported sleep quality in future trials of VMS treatments.

Menopausal hormone therapy is currently the most effective treatment for VMS associated with menopause [11]. However, many women in both the United States and European Union cannot or choose not to take hormone therapy [11–13]. Fezolinetant is an oral nonhormonal neurokinin 3 receptor antagonist currently in development for treatment of VMS associated with menopause [14, 15]. It is hypothesized to act by directly modulating the activity of a subset of hypothalamic neurons that are believed to play a key role in thermoregulation [14–16]. Fezolinetant significantly reduced both the severity and frequency of moderate to severe VMS and was well tolerated in two phase 2 clinical trials enrolling postmenopausal women with moderate or severe VMS [14, 15]. In one of the investigations, a phase 2a trial, results also reflected that treatment with fezolinetant improved sleep quality based on the Leeds Sleep Evaluation Questionnaire [14]. The Leeds Sleep Evaluation Questionnaire was developed to assess adverse effects of medications on sleep [17] and may or may not assess concepts important to women with VMS associated with menopause. As sleep impact is an important area for study, two more appropriate and robustly developed PROMIS measures—Patient-Reported Outcome Measurement Information System (PROMIS) Sleep-Related Impairment Short Form 8a (PROMIS SRI SF 8a; Table 1) [18] and PROMIS Sleep Disturbance Short Form 8b (PROMIS SD SF 8b; Table 2) [19]—were included as sleep assessment measures in the fezolinetant phase 3 trials, which are now under way.

**Table 1:**
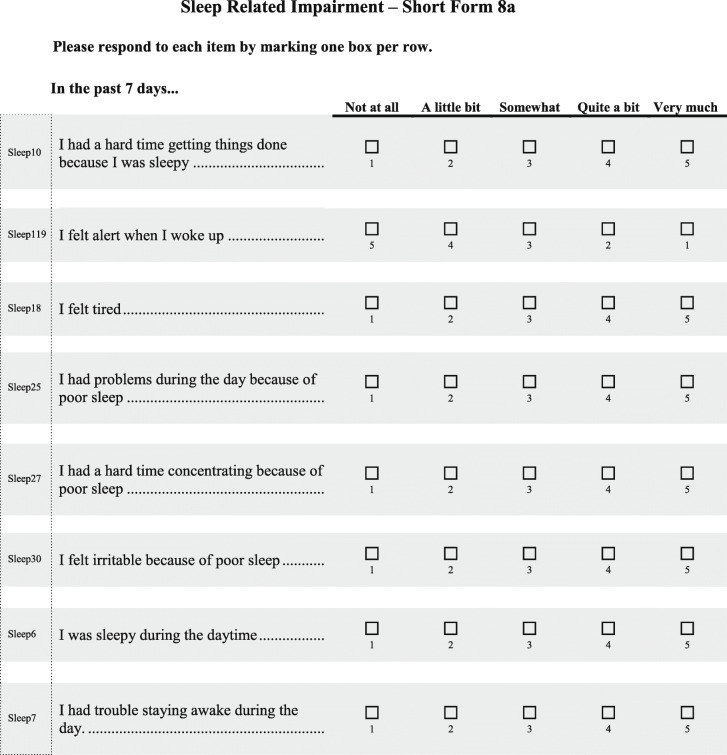
PROMIS Sleep-Related Impairment Short Form 8a [18]

Reproduced with permission from Health Measures (00030669). PROMIS measures are available at: http://www.healthmeasures.net/explore-measurement-systems/promis/obtain-administer-measures

**Table 2:**
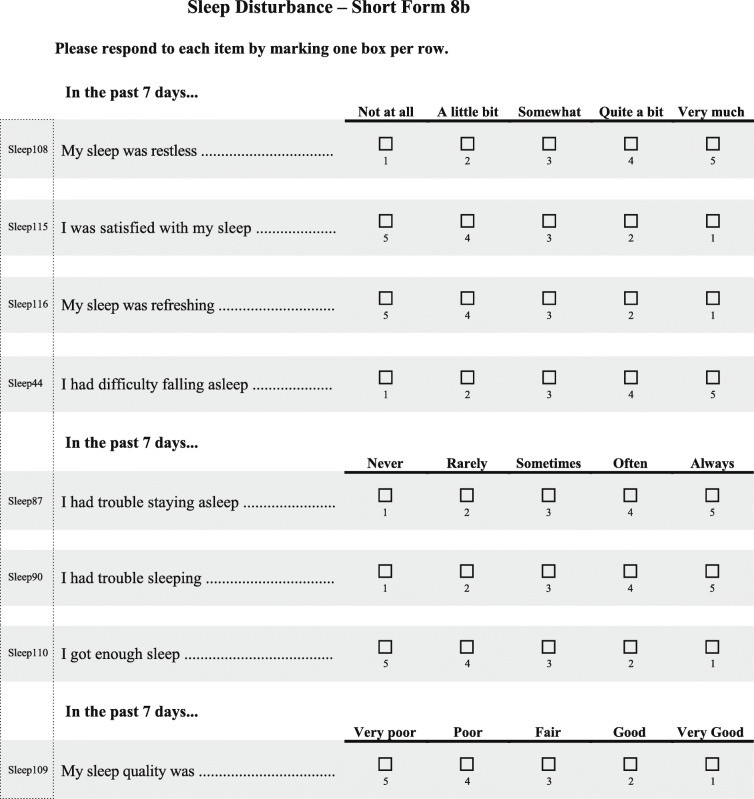
PROMIS Sleep Disturbance Short Form 8b [19]

Reproduced with permission from Health Measures (00030669). PROMIS measures are available at: http://www.healthmeasures.net/explore-measurement-systems/promis/obtain-administer-measures

PROMIS measures were developed by the US National Institutes of Health to be universally relevant across the general population and in individuals with a range of chronic diseases [20, 21]. During development, long- and short-form versions of the two PROMIS sleep measures were tested in the general population and those with sleep disorders [22, 23] but not specifically in menopausal women with VMS. Therefore, content validity in women with sleep difficulties related to VMS associated with menopause has not yet been established.

According to the International Society for Pharmacoeconomics and Outcomes Research (ISPOR), the content validity of a PRO instrument should be established by qualitative analysis “by documenting that the structure and content (items) [of the PRO] capture the connection between the intended measurement concept and the way patients from the target population understand and discuss that concept” [24]. COSMIN methodology specifies that content validity of a PRO should be based on relevance, comprehensiveness, and comprehensibility [25]. Further, the US Food and Drug Administration (FDA) provides guidance for the evaluation of a PRO instrument for use as an endpoint to measure treatment benefit in clinical trials [26]. In accordance with the recommendations outlined in that guidance document, a qualitative study was designed to assess the suitability of PROMIS SRI SF 8a and PROMIS SD SF 8b for evaluating the potential effect of fezolinetant on sleep disruptions related to moderate to severe VMS in menopausal women participating in phase 3 trials.

We conducted a cross-sectional qualitative study in menopausal women experiencing moderate to severe VMS. The first objective was to obtain a better understanding of women’s experiences with VMS, including their symptoms and impacts, especially impact on sleep. The second objective was to evaluate the content validity of PROMIS SRI SF 8a and PROMIS SD SF 8b measures in menopausal women with moderate to severe VMS and confirm they are fit-for-purpose in measuring impacts of menopause-associated VMS on sleep.

## Methods

### Participant selection and recruitment

In-person interviews were conducted with women 40–64 years of age (inclusive) who were experiencing ≥35 moderate to severe hot flashes and/or night sweats per week. Moderate VMS were defined as sensations of heat with sweating without disruption of activity; severe VMS were defined as sensations of heat with sweating prompting cessation of normal activity. To be eligible, women also had to have either undergone removal of both ovaries ≥6 weeks prior to screening or had no menstruation for ≥12 months prior to screening due to menopause or treatment for breast cancer (e.g., tamoxifen). Exclusion criteria included pregnancy; cessation of menses due to contraceptive use, pregnancy/breastfeeding, and/or treatment for a medical condition other than breast cancer; and current malignancy, with the exception of breast cancer currently treated with maintenance hormonal therapy.

Convenience samples were used in all countries. Materials developed by RTI Health Solutions (RTI-HS; Research Triangle Park, NC) and Astellas were used to recruit and screen potential interview participants. At each location, medical recruiters from RTI-HS’s partnering research firms used their own proprietary databases to identify and contact via telephone or email an appropriate sample of individuals who had expressed interest in participating in qualitative research and/or individuals who had previously participated in similar research projects. As needed, medical recruiters also posted advertisements on their facilities’ websites and on social media to assist recruitment efforts. In the European Union, recruiters also advertised the research project to patient support groups; women who were interested in participating contacted the recruiters and were screened for eligibility. Participants provided written informed consent prior to the start of the interviews.

### Interviews

Sixty-minute interviews were conducted at a local qualitative research facility, office, or hotel in the United States (Dallas, TX; Chicago, IL) or European Union (Manchester, UK; Paris, France). One southern and one northern US city (Dallas, TX, and Chicago, IL) were chosen to represent a range of climates.

Each woman participated in a single interview conducted in two phases using a semistructured Interview Guide (described in **Online Resource** **1****)**. The first phase consisted of concept elicitation using a “think aloud” technique, which began with open-ended questions to assess the participant’s experiences with and perspective of the burden of VMS, followed by scripted probing questions to ensure discussion of key concepts of interest (e.g., impact of VMS on sleep and daily activities) that may not have been covered by the open-ended questions. The second phase consisted of cognitive debriefing to assess the content validity of the PROMIS SD SF and SRI SF: participants were asked to complete the two measures; give their overall impression; and comment on the relevance, importance, and clarity of the items, questions, and response scales. Participants were also asked whether the PROMIS measures were comprehensive. Women in the United States were interviewed until concept saturation was reached (i.e., until no new relevant knowledge was being generated) during concept elicitation. Interviews in the United Kingdom and France were subsequently conducted to confirm results obtained in the US sample, so concept saturation was reached at a smaller sample size.

US and UK interviews were conducted in English with English-speaking participants. Interviews in France were conducted in French by a native French speaker with translated study materials; official French translations of the two PROMIS sleep measures were used, and additional interview materials (e.g., recruitment materials, informed consent, interview guide) were translated and proofread by a partnering independent research firm with extensive experience conducting qualitative interviews (AplusA, Paris, France). The interviews were audio recorded and transcribed for analysis, and transcripts of the French interviews were translated into English.

### Research team

US and UK interviews were conducted by two female teams from RTI-HS, each with extensive qualitative experience in women’s health conditions, including menopause (authors DD and CS in the United States, and LD and RC in the United Kingdom). The interview teams reviewed the Interview Guide prior to the conduct of interviews in each country to ensure that the interview process and data collection were consistent across locations. One person served as the primary interviewer while the other took notes and monitored the need for additional questions/probes. French interviews were conducted by a female native French speaker from APlusA.

### Data analyses

Data analyses were conducted by RTI-HS. All English transcripts were coded according to a prespecified qualitative analysis plan that included a preliminary code book based on the final interview guide. Additional codes were added during analysis to incorporate any emerging themes. Concept saturation was considered to be reached when no new codes were identified during successive transcript sets. Most of the coding was done by a single coder (author CS); however, as a quality control measure, about 10% of transcripts (four) were double coded to check consistency; as no problems were found, no further double coding was conducted. Any discrepancies were resolved by the two coders in discussion with the lead US interviewer. ATLAS.ti software version 7.5 or higher and Microsoft Excel were used to facilitate the analysis.

Analyses were performed using descriptive statistics for continuous variables and frequency and percentages for categorical variables.

## Results

### Participants

A total of 32 women participated (US: *n* = 16; EU: n = 16); all completed the interview process. Interviews were conducted with 9 women in Dallas, TX (March 12–13, 2018); 7 women in Chicago, IL (March 26–27, 2018); 8 in Manchester, UK (September 5–6, 2018); and 8 in Paris, France (February 14–27, 2019).

The US and EU participants were similar in age (Table 3). All had experienced natural or surgical menopause; none had cessation of menses resulting from treatment for breast cancer. There were more non-white participants in the United States (43.75%), whereas all 8 participants were white in the United Kingdom; collection of data on race was not permitted in France. More US than EU participants had undergraduate or graduate degrees; obesity, hypertension, diabetes, or a family history of breast cancer; and current or prior exposure to menopausal hormonal therapy (Table 3). US and EU participants were similar with regard to age at VMS onset, but the women from the European Union had a slightly higher mean number of moderate and severe VMS per week (Table 4). Of the 16 women in the United States, 15 reported experiencing moderate and 14 reported severe VMS events, and of the 16 women in the European Union, 13 reported moderate and 15 reported severe VMS events.

**Table 3:** Demographic and clinical characteristics

	US Participants			EU Participants		
	Dallas, TX (***n*** = 9)	Chicago, IL (***n*** = 7)	Total US (***N*** = 16)	UK (***n*** = 8)	France (***n*** = 8)	Total EU (***N*** = 16)
Age, mean (SD), y	55.6 (4.3)	56.4 (3.0)	**55.9 (3.7)**	53.3 (6.5)	57.4 (4.5)	**55.3 (5.8)**
Race/ethnicity, *n* (%)						
White	5 (55.6)	4 (57.1)	**9 (56.3)**	8 (100)	ND^a^	**ND**^**a**^
Black	3 (33.3)	3 (42.9)	**6 (37.5)**	0	ND^a^	**ND**^**a**^
Hispanic	1 (11.1)	0	**1 (6.3)**	0	ND^a^	**ND**^**a**^
Highest education level						
High school/GED/secondary school	1 (11.1)	0	**1 (6.3)**	3 (37.5)	5 (62.5)	**8 (50.0)**
Some college	3 (33.3)	2 (28.6)	**5 (31.5)**	–	–	–
Associates degree (US) or vocational/technical qualifications completed (EU)	1 (11.1)	0	**1 (6.3)**	3 (37.5)	0	**3 (18.8)**
Undergraduate degree	3 (33.3)	2 (28.6)	**5 (31.3)**	1 (12.5)	2 (25.0)	**3 (18.8)**
Some graduate school	1 (11.1)	0	**1 (6.3)**	–	–	–
Graduate/professional degree (US) or postgraduate qualification (EU)	0	3 (42.9)	**3 (18.8)**	1 (12.5)	1 (12.5)	**2 (12.5)**
BMI, n (%)						
< 25 kg/m^2^	2 (22.2)	2 (28.6)	**4 (25.0)**	2 (25.0)	3 (37.5)	**5 (31.3)**
25.0–29.99 kg/m^2^ (overweight)	1 (11.1)	2 (28.6)	**3 (18.8)**	2 (25.0)	2 (25.0)	**4 (25.0)**
30.0–34.99 kg/m^2^ (obese class 1)	2 (22.2)	2 (28.6)	**4 (25.0)**	4 (50.0)	2 (25.0)	**6 (37.5)**
35.0–39.99 kg/m^2^ (obese class 2)	3 (33.3)	1 (14.3)	**4 (25.0)**	0	1 (12.5)	**1 (6.3)**
≥ 40.0 (obese class 3)	1 (11.1)	0	**1 (6.3)**	0	0	**0**
Comorbid conditions						
Hypertension	2 (22.2)	2 (28.6)	**4 (25.0)**	0	2 (25.0)	**2 (12.5)**
Mental health condition	3 (33.3)	0	**3 (18.8)**	2 (25.0)	1 (12.5)	**3 (18.8)**
Elevated cholesterol	2 (22.2)	1 (14.3)	**3 (18.8)**	2 (25.0)	1 (12.5)	**3 (18.8)**
Type 2 diabetes	1 (11.1)	1 (14.3)	**2 (12.5)**	0	0	**0**
Family history of breast cancer	3 (33.3)	3 (42.9)	**6 (37.5)**	1 (12.5)	1 (12.5)	**2 (12.5)**
Stroke/TIA	0	0	**0**	1 (12.5)	0	**1 (6.3)**
Type of menopause						
Natural	8 (88.9)	7 (100)	**15 (93.8)**	8 (100)	8 (100)	**16 (100)**
Surgical^b^	1 (11.1)	0	**1 (6.3)**	0	0	**0**
Cessation of menses due to breast cancer treatment	0	0	**0**	0	0	**0**
HT use, *n* (%)						
Current user	1 (11.1)	0	**1 (6.3)**	0	0	**0**
Previous user	3 (33.3)	3 (42.9)	**6 (37.5)**	0	2 (25.0)	**2 (12.5)**
Never used	5 (55.6)	4 (57.1)	**9 (56.3)**	8 (100)	6 (75.0)	**14 (87.5)**

^a^Collection of race/ethnicity data was not permitted in France

^b^Bilateral oophorectomy, including hysterectomy

*BMI* body mass index, *GED* General Educational Development test, *HT* hormonal therapy, *ND* not determined, *SD* standard deviation, *TIA* transient ischemic attack

**Table 4:** Menopausal symptoms reported at screening

	US Participants			EU Participants		
	Dallas, TX (***n*** = 9)	Chicago, IL (***n*** = 7)	Total US (***N*** = 16)	UK (***n*** = 8)	France (***n*** = 8)	Total EU (***N*** = 16)
Age at VMS onset, mean (SD), y	46.2 (6.2)	49.9 (5.3)	**47.8 (5.9)**	48.3 (4.4)	48.9 (4.0)	**48.6 (4.1)**
Average weekly number of moderate to severe VMS,^a^ mean (SD)						
Moderate	34.1 (15.2)	27.1 (13.9)	**30.9 (14.5)**	40.3 (19.0)	25.0 (28.7)	**32.6 (24.8)**
Severe	27.2 (23.3)	31.7 (27.7)	**29.2 (24.5)**	25.4 (22.7)	44.1 (21.4)	**34.8 (23.4)**
Other menopausal symptoms at screening,^b^ *n* (%)						
Problems sleeping	8 (88.9)	7 (100)	**15 (93.8)**	6 (75.0)	6 (75.0)	**12 (75.0)**
Mood swings	6 (66.7)	2 (28.6)	**8 (50.0)**	6 (75.0)	6 (75.0)	**12 (75.0)**
Weight gain	7 (77.8)	7 (100)	**14 (87.5)**	5 (62.5)	6 (75.0)	**11 (68.8)**
Vaginal dryness	6 (66.7)	4 (57.1)	**10 (62.5)**	4 (50.0)	4 (50.0)	**8 (50.0)**
Memory/concentration problems	5 (55.6)	3 (42.9)	**8 (50.0)**	7 (87.5)	0	**7 (43.8)**
Headaches	4 (44.4)	3 (42.9)	**7 (43.8)**	5 (62.5)	2 (25.0)	**7 (43.8)**
Depression	3 (33.3)	1 (14.3)	**4 (25.0)**	1 (12.5)	0	**1 (6.3)**

*VMS* vasomotor symptoms

^a^Participants were asked at screening, “In a typical week, how many hot flashes (including night sweats) do you have that are mild, moderate, or severe?” and were asked to provide the “number per typical week” for each severity level. Participants were provided with the following definitions: mild = sensation of heat without sweating; moderate = sensation of heat with sweating, but able to continue activity; severe = sensation of heat with sweating, causing cessation of activity

^b^Participants were queried at screening as to whether they had each of the listed symptoms

During screening, participants were asked if they had specific symptoms of menopause, including mood swings, problems sleeping, headaches, memory problems/trouble thinking or concentrating, vaginal dryness, depression, and weight gain. A majority of participants reported having sleep problems (US: 93.8%; EU: 75.0%), which were the most common of these symptoms reported, although mood swings were reported by a similarly high percentage of women in the European Union (75%; Table 4).

### Concept elicitation

#### Women’s experiences with VMS

US participants reported experiencing VMS for the past 2 to 23 years (mean: 8.1 years), and EU participants for the past 2 to 16 years (mean: 5.5 years). VMS were the first menopausal symptoms experienced by 9 (56.3%) US participants and 13 (81.3%) EU participants. Most US and EU participants reported VMS lasting typically from a few seconds to a few minutes, but three US women reported VMS lasting 15 to 30 min.

US women described hot flashes as coming in “waves” or a building sense of pressure and accompanied by sweating that was like “a downpour” or “a faucet.” EU women focused on the sudden nature and intensity of the heat associated with VMS, and many reported feeling embarrassed by visible flushing and sweating. Representative quotations are reported in **Online Resource** **1**. Across all samples, common strategies for coping with VMS included wearing less clothing, layers, or breathable clothing; using a fan or air conditioner; and drinking cold water (Table 5).

**Table 5:** Representative quotations from concept elicitation phase on sleep impacts and self-care

	US Participants	EU Participants
**Sleep Impacts and Related Self-Care**	“You have to either change the pillow or turn it over. Many times I have 4 pillows at night because … I knew that if I get so wet, I would just change the pillow.” –Dallas-1 “It got so bad to where I had to get in the shower at night and go back to bed. It was just a gross feeling.” –Dallas-7 “I sleep in a short-sleeve gown that I take off in the course of the night. I still wear socks…but in the course of the night… I may take one off because my body is so hot.” –Chicago-2 “My husband and I have to sleep in separate rooms because I have to have the fan.” –Chicago-4 “[B]eing sleep deprived touches so many other aspects of being able to function the next day … and then you can’t catch up on it. You never recoup that loss.” –Chicago-7	“I feel like … I’m sleeping in a kettle.” –France-6 “… last night I was a bit chilly, so its typical duvet on, duvet off, you know, through the night… just a few hours of really good sleep.” –UK-4 “The hot flash itself goes away rather quickly but the feeling of warmth and heat remain longer. It means that if I want to go back to sleep peaceful, it will take at least 20 minutes” –France-2 “I am waking up nearly every hour on the hour … I don’t think I have had a decent night’s sleep for about 6 months.” –UK-7 “What bothers me most today are the hot flashes and the fact that I do not sleep properly anymore … that makes me suffer.” –France-6 “I have three or four pillows so they remain cool … I keep switching between them … Even if it is −15 [degrees] outside, my window is open.” –France-1 “I was waking him all night really. I would suddenly say I am hot and I would throw the quilt off and of course my husband would wake up then as well, so I moved into a different bedroom.” –UK-1
**Daytime Self-care Impacts and Strategies for Coping With VMS**	“I turn the air conditioner on in the wintertime or a fan.” –Chicago-4 “I tend to dress in layers.” –Chicago-6 “If I’m going somewhere … I wait until the last minute before I get dressed.” –Dallas-4 “… you get so sweaty and you need to change your [underwear]. And how do you explain you need to change your panties? Like I’m changing a diaper.” –Dallas-1	“I have always got spare clothes at work in case I have like a really bad hot flush.” –UK-5 “I’ll take a shower … and then I’ll get my hot flash so I sweat… then I shower again.” –France-5 “I am always conscious of sweaty smell … and I think if your body is perspiring anyway you are worried about the smell.” –UK-8 “I always have tissues on me … I know that I have to wipe off … especially in public transportation.” –France-5

#### Sleep impacts of VMS

**Fig. 1:**
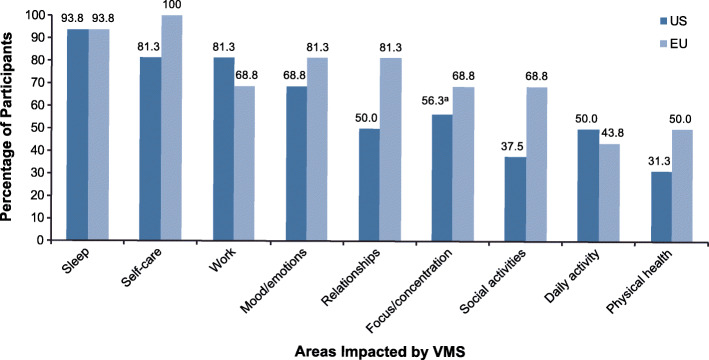
Areas of life impacted by VMS associated with menopause. During the concept elicitation phase, women were asked general, open-ended questions about the way VMS impacted their lives, and then asked how VMS impacted each of the specific areas shown here. Responses are categorized here to show the percentage of participants who indicated that each area is impacted by their VMS. The EU sample included participants in Paris, France, and Manchester, UK; the US sample included participants in Dallas, TX, and Chicago, IL. ^a^Includes 5 women who reported difficulty concentrating during hot flashes and 4 who reported that lack of sleep due to VMS made them less alert or focused the following day. VMS, vasomotor symptoms

During the concept elicitation portion of the interview, a majority of the women (US: 93.8%; EU: 93.8%) mentioned impacts of VMS on sleep (Fig. 1). While participants generally did not have difficulty falling asleep initially, VMS commonly resulted in sleep interruptions, owing to physical discomfort (profuse sweating, overheating), and subsequent difficulty returning to sleep. Participants reported waking up several times during a typical night and feeling hot, wet, or both. Night sweats led women to lift covers on and off during sleep or switch to a cooler or drier pillow. Some women woke up so drenched with sweat they needed to remove nightclothes, change their bedding, get a glass of water or cool towel, or get up to shower (Table 5). Sleep disturbance was considered the most bothersome effect of VMS by 75% of the US women and 50% of the EU women (Table 6).

**Table 6:** Most bothersome impacts of menopausal VMS

Most bothersome impact, ***n*** (%)	US Participants			EU Participants		
	Dallas, TX (***n*** = 9)	Chicago, IL (***n*** = 7)	Total US (***N*** = 16)	UK (***n*** = 8)	France (***n*** = 8)	Total EU (***N*** = 16)
Sleep	6 (66.7)	6 (85.7)	**12 (75.0)**	4 (50.0)	4 (50.0)	**8 (50.0)**
Discomfort^a^	1 (11.1)	1 (14.3)	**2 (12.5)**	3 (37.5)	4 (50.0)	**7 (43.8)**
Social activities	0	0	**0**	1 (12.5)	0	**1 (6.3)**
Relationship with partner	1 (11.1)	0	**1 (6.3)**	0	0	**0**
Morning routine	1 (11.1)	0	**1 (6.3)**	0	0	**0**

*VMS*, vasomotor symptoms

^a^Discomfort includes feeling sweaty or hot, and subsequent irritability

#### Other VMS impacts

VMS had impacts on work, self-care, and mood/emotions for a majority of US and EU women (Fig. 1). Representative quotations are reported in **Online Resource** **2**. Work productivity was affected by interruptions due to needing to cool off. VMS prompted many women to more frequently change clothes, shower, and apply deodorant/perfume. Some also reported having to wash or change their bedding more often. VMS caused emotional or mood disturbances; women reported becoming irritable, overly emotional, uncomfortable, and embarrassed in both social and professional settings by visible symptoms or odors.

VMS also had an impact on relationships, social activities, and focus/concentration for a majority of the EU women and nearly 60% of the US women (Fig. 1). Both EU and US women reported that VMS adversely affected relationships with coworkers and family members, especially their spouses/partners. In particular, VMS caused some women to dislike being touched and to restrict physical intimacy with their partners; 5 participants (1 in the EU and 4 in the US) noted they slept in separate rooms from their partners because of the need to keep the room cooler or to avoid disturbing the partner by adjusting the bed covers during the night. In the United States, 62.5% of women said they do not allow VMS to affect their social lives or prevent them from socializing, whereas in the European Union, more than two thirds of women reported some restriction on their social activities, including changes to the types of events they would attend or time of year to take vacations. The physical sensation of hot flashes and related daily interruptions and sleep impairments were noted to interfere with memory or focus and concentration in both groups (EU: 69%; US: 56%).

VMS had an impact on daily activities for half of the US participants and a slightly smaller proportion (44%) of EU participants (Fig. 1). Those who were affected noted being slower or struggling more to complete tasks and felt tired during the day owing to inadequate sleep related to VMS. Physical health was affected for half of EU participants and nearly one third of US participants (Fig. 1). Those affected said hot flashes were sometimes accompanied by feeling nauseous or dizzy, ringing in the ears, feeling physically drained, or feeling like their blood pressure had increased. One woman said VMS were triggers for severe headaches.

#### Cognitive debriefing: content validity of the PROMIS sleep measures

All participants found both PROMIS sleep measures (**Tables** **1** and **2**) easy to understand and answer and relevant to VMS’ impact on sleep (Table 7). The two measures were found to capture different but relevant aspects of the impact of VMS on sleep. Participants did not have difficulty remembering their experiences over the 7-day recall period. EU participants said they generally averaged their sleep time from the past 7 nights when responding, although a few of the EU women responded based on their worst night’s sleep during the past week. Women in both the United States and European Union believed their responses to the questions would change if their symptoms improved, and they generally found the response options to be distinct and were able to select from among them. Although a few French participants said they would have preferred the items to be more specific in terms of the underlying cause of the sleep difficulties (VMS- vs non–VMS-related), participants were able to provide responses to all items as they are currently written.

**Table 7:** Representative quotations from the cognitive debriefing phase related to PROMIS SRI SF 8a (Sleep-Related Impairment) and PROMIS SD SF 8b (Sleep Disturbance)

	US Participants	EU Participants
**Overall assessments of the PROMIS scales**	“They fit night sweats and menopause. I mean they are very good.” –Dallas-7 “They were easy to understand.” –Dallas-6	“I really love [the] form[s], as it is clear. It is well-defined. It is comprehensible.” –France-6 “I did not find any great difficulty [recalling the past 7 days]. At first, you have to put yourself back into the situation and look back at the 7 past days. It simply requires a few seconds to remember.” –France-2
**PROMIS response choices**	“I really like [the response options] because it gives you quite a bit to choose from … you’re not limited to just ‘not at all’ and ‘very much’.” –Dallas-7	“There are five possible answers and women can find themselves somewhere on that scale. I really liked that.” –France-6
**Differentiating the two PROMIS scales**	“They cover two different areas. The actual side of the sleeping quality of sleep [SF 8b] and then the consequences after a bad night [SF 8a].” –Dallas-1	“I would say both [should be included in a trial] because that one [SF 8b] asks you about the sleep cycle, going to sleep, staying asleep, falling asleep, and that one [SF 8a] is how did you feel that day after it all happened.” –UK-2
**PROMIS SRI SF 8a (Sleep-Related Impairment)**	“Some things you don’t think about, you just move through it … so when you look at it on paper and I think about it … it’s kind of scary … that you’re functioning at a deficit almost.” –Chicago-7	“For me, [PROMIS SRI SF 8a is] a fair reflection looking back on it, how [hot flashes and night sweats] affect me.” –UK-5 “Sometimes the link with hot flashes is not obvious. I was sleepy during the daytime. Everyone can get drowsy during the day, without it meaning that it is necessarily linked to the hot flashes.” –France-4
**PROMIS SD SF 8b (Sleep Disturbance)**	“It’s trouble staying asleep I put ‘often’ … that’s probably for me the most difficult thing.” –Chicago-2 “So you won’t have trouble falling asleep. It’s the maintaining of how you [sleep]…it’s the falling part that I have some reservations about because I can fall asleep. It’s just I’m losing the sleep with the flashes.” –Dallas-8	“The questions were well targeted, so I was able to answer them without any problem.” –France-6 “Probably sleep was restless, trouble sleeping [would change the most with a treatment]. If I manage to just go back to sleep, I still feel okay, but if it has been a night where I have woken up a lot, and it has taken a while to go back to sleep, I have probably not had enough then, so I am still tired.” –UK-1

#### PROMIS SRI SF 8a (Sleep-Related Impairment)

Based on responses on the PROMIS SRI SF 8a, more than half of US participants said VMS had an impact ranging from “a little bit” to “very much” on alertness, tiredness, daytime problems due to poor sleep, and daytime sleepiness. Half of EU participants said that VMS at least “somewhat” affected alertness, concentration, irritability, and ability to get things done because of sleepiness.

Across the 3 countries, participants generally agreed that the PROMIS SRI SF 8a captured the negative daytime consequences of poor sleep, which was considered an important concept to this sample. Because the measure does not specifically ask participants to think about VMS, 5 of the EU women said they considered all menopausal symptoms and other factors when answering the questions. A majority of EU participants felt that items 7 (“I was sleepy during the daytime”) and 8 (“I had trouble staying awake during the day”) were repetitive, but they were still able to answer both items without difficulty.

Participants in this study found the PROMIS SRI SF 8a fairly comprehensive overall. A couple of participants suggested adding items related to effects of sleep impairment on next-day emotions (US: *n* = 1; EU: n = 1) and energy levels (EU: *n* = 2).

#### PROMIS SD SF 8b (Sleep Disturbance)

The women we interviewed agreed that the PROMIS SD SF 8b measure focused on important aspects of the sleep disturbances they experienced. Items considered most affected by VMS were “trouble staying asleep” and “sleep quality was restless.”

In the United States, 10 participants (62.5%) reported at least “somewhat/sometimes/fair” disturbance on items 1 (restless sleep), 2 (sleep satisfaction), 3 (refreshing sleep), 5 (trouble staying asleep), and 8 (sleep quality) and said their answers would change most on these items if their VMS improved. Item 5 was considered most affected by VMS. The order of the first four items was confusing to some of the US participants because the items switched from negative to positive and back to negative phrasing; however, participants were able to select appropriate responses. In the European Union, 10 women (62.5%) reported at least “somewhat/sometimes/fair” disturbance on six of the eight items, with item 1 (“sleep quality was restless”) considered most affected by VMS.

Both US and EU participants also found the PROMIS SD SF 8b to be comprehensive. US and EU participants noted redundancy between item 5 (trouble staying asleep) and item 6 (trouble sleeping) yet, again, were able to answer each item. Overall, PROMIS SD SF 8b was considered the more relevant of the two measures because it could directly capture changes in sleep that resulted from VMS, whereas the PROMIS SRI SF 8a measure was more indirect in that it focused on next-day impacts of having slept poorly.

## Discussion

There are several medical conditions that impact quality of sleep (eg, nocturia associated with benign prostatic enlargement, overactive bladder, diabetes mellitus), and as a consequence, they negatively impact quality of life. However, the impact on sleep is not always measured using standardized and validated tools. Similarly, there is growing appreciation of the impact of VMS associated with menopause on sleep quality and a need for standardized, validated sleep questionnaires to evaluate sleep outcomes in future trials of VMS therapies. This study examined the burden of menopause-associated VMS and the validation of PROMIS sleep disturbance and sleep-related impairment measures for assessment of VMS impact on sleep.

In this research study, women with moderate to severe VMS associated with menopause reported that these symptoms had a significant impact on their lives, especially their sleep. Based on both the concept elicitation phase and responses on the PROMIS measures, reported sleep difficulties included nocturnal awakenings due to feeling hot or sweaty, restless sleep, unrefreshing sleep, dissatisfaction with sleep, and poor sleep quality. All participants reported that the PROMIS sleep measures would provide an accurate evaluation of their current sleep disturbances and sleep-related impairments, and no major issues or concerns were identified with these measures. Results were largely consistent between US and EU interviews. Both PROMIS sleep measures assessed concepts that aligned with those endorsed by the women as being relevant and important during the concept elicitation phase.

Results of our qualitative research indicate that both the PROMIS SRI SF 8a and PROMIS SD SF 8b are useful measures for assessment of sleep-related impairments and sleep disturbances, respectively, in women with moderate to severe VMS associated with menopause. Participants considered PROMIS SD SF 8b to be the more relevant of the two measures. The current findings thus suggest that the PROMIS SD SF 8b will be an effective measure for assessing women’s qualitative experience of sleep difficulties associated with VMS, as opposed to quantitative (e.g., sleep duration, sleep onset latency) aspects of sleep [22, 23].

According to FDA guidance, a PRO instrument used in clinical trials should be shown to measure the concept it is intended to measure in the population in which it will be used [26]. Demonstration of concept validity for PRO instruments requires that all items, domains, and general scores reflect what is important to patients and are comprehensive with respect to patient concerns relevant to the concept being assessed. It is also important that patients be able to recall the information requested over the duration of the recall period (e.g., 7 days in the case of the PROMIS sleep measures). Wording and instructions should be clear and appropriate, responses should be suitable for the population, appropriately ordered, and offer distinct options without biasing direction of the response. Based on results of the current analysis, the two PROMIS SF sleep measures assessed here meet these FDA criteria.

COSMIN criteria for content validity of a PRO require that the measure be relevant, comprehensive, and comprehensible [25]. The women who participated in our interviews were representative of the target population of women with moderate to severe VMS associated with menopause from multiple geographic regions. They consistently found the two PROMIS measures to be relevant to their experiences with sleep impacts of VMS and said the response options and recall period were appropriate. Participants felt the measures were reasonably comprehensive, covering the major impacts that VMS have on their sleep and next-day functioning. They were able to understand the instructions, items, and response options and were able to answer all of the questions. They felt the questions and response options were appropriate. Thus, the COSMIN criteria were fulfilled.

We concluded that since the two PROMIS SF sleep measures assessed here meet the FDA and COSMIN criteria, they could be used in future trials assessing sleep interference and sleep disturbance related to VMS in menopausal women with moderate to severe symptoms. Therefore, the PROMIS SD SF 8b measure was selected for use as a secondary outcome measure in phase 3 fezolinetant trials, which will gather placebo-controlled data in a large population of women with VMS associated with menopause. PROMIS SRI SF 8a, which addresses the daytime consequences of those sleep disturbances, was included as an exploratory supportive measure in phase 3 fezolinetant studies.

A key strength of the current analysis is that we used a multinational sample large enough to confirm the relevance of concepts of interest and demonstrate replicability across different geographic regions. The interview format was consistent with regulatory and industry standards [26, 27], and the analysis was conducted in accordance with guidance from ISPOR Task Force Reports for establishing content validity of PROs [24, 28, 29]. The interviews allowed for collection of broad, in-depth information, and the use of a semistructured interview guide ensured that data were collected in a consistent and systematic manner while encouraging spontaneous responses and a conversational tone. The flexible format allowed further probing to generate appropriately detailed information. One-on-one interviews allow for privacy and confidentiality, which may encourage greater openness than can be obtained with focus groups.

A number of limitations should also be acknowledged. The relatively small number of participants and convenience sampling may limit generalizability; however, concept saturation was reached such that adding participants was believed unlikely to generate additional concepts regarding sleep patterns in this population. Previous research has shown that 6 to 12 interviews can be sufficient to provide saturation of thematic content [30], and 8 to 12 interviews are usually sufficient to identify any major issues with questionnaires before amending them, if necessary, and beginning another round [31]. We cannot rule out the possibility of bias in the patient population selected or the potential for implicit bias or influence on the part of the interviewers who engaged closely with participants. Eligibility was determined by self-reported responses to the screening questionnaire and was therefore subject to the limitations of participant recall. Some participants may have felt uncomfortable sharing personal details about their experiences with an unknown interviewer, although the women seemed to be generally forthcoming about the impacts of VMS on their lives. In addition, consistent with most clinical trial populations, women in the current study had to be experiencing moderate to severe VMS at study entry, which may limit the generalizability to women with mild symptoms.

## Conclusion

Menopausal women with moderate to severe VMS reported that VMS resulted in frequent nighttime awakenings owing to unpleasant sensations of heat and sweating. Many women reported the need for intensive coping strategies and self-care activities related to both daytime and nighttime VMS. Sleep disturbances due to VMS affected women’s ability to focus and function the next day. While VMS had a wide range of impacts, particularly in areas such as work, self-care, emotions, and relationships, sleep disruption was considered the most bothersome area affected, supporting the assessment of sleep outcomes in clinical trials of treatments for VMS associated with menopause.

Women with moderate to severe VMS associated with menopause found the questions from the PROMIS SRI SF 8a and PROMIS SD SF 8b measures easy to answer, and said they assessed constructs relevant to the day-time consequences of disrupted sleep and the sleep disturbances related to VMS that give rise to them. PROMIS SD SF 8b was considered the more relevant of the two measures. Results were generally consistent between the US and EU survey responses, especially regarding feedback on the PROMIS measures. Thus, content validity was broadly established, and PROMIS SRI SF 8a and PROMIS SD SF 8b measures were confirmed to be fit for purpose without modification.

## Supplementary Information

**Additional file 1: Online Resource 1**. Description of Semi-Structured Interview Guide. **Online Resource 2**. Additional Representative Quotations

## Data Availability

Researchers may request access to anonymized participant-level data, trial-level data, and protocols from Astellas-sponsored clinical trials at www.clinicalstudydatarequest.com. For the Astellas criteria on data sharing see: https://clinicalstudydatarequest.com/Study-Sponsors/Study-Sponsors-Astellas.aspx.
