# Correction to: Qualitative study: burden of menopause-associated vasomotor symptoms (VMS) and validation of PROMIS sleep disturbance and sleep-related impairment measures for assessment of VMS impact on sleep

**DOI:** 10.1186/s41687-021-00322-0

**Published:** 2021-06-01

**Authors:** Marci English, Boyka Stoykova, Christina Slota, Lynda Doward, Emad Siddiqui, Rebecca Crawford, Dana DiBenedetti

**Affiliations:** 1 Astellas Pharma Inc., Pharma Global Development, Astellas Way, Northbrook, IL 60062–6111 USA; 2 Astellas Pharma Inc., Chertsey, Surrey UK; 3 RTI Health Solutions, Patient-Centered Outcomes Assessment Group, Research Triangle Park, NC USA; 4 RTI Health Solutions, Manchester, UK

**Correction to: Journal of Patient-Reported Outcomes 5, 37 (2021).**

**https://doi.org/10.1186/s41687-021-00289-y**

After publication of the original article [1], an error was identified in both Tabl1 and 2.

The below footnote should be added:

“Reproduced with permission from Health Measures (00030669). PROMIS measures are available at: http://www.healthmeasures.net/explore-measurement-systems/promis/obtain-administer-measures”.

The original article has been corrected.
