# Supplementary material for: Qualitative study: burden of menopause-associated vasomotor symptoms (VMS) and validation of PROMIS Sleep Disturbance and Sleep-Related Impairment measures for assessment of VMS impact on sleep
Source: J Patient Rep Outcomes. 2021 Apr 26;5:37. doi: 10.1186/s41687-021-00289-y (PMC8076383; doi:10.1186/s41687-021-00289-y)
Supplement: Supplementary file 1 — Additional file 1: Online Resource 1. Description of Semi-Structured Interview Guide. Online Resource 2. Additional Representative Quotations [file 41687_2021_289_MOESM1_ESM.docx]

**ONLINE RESOURCES**

**Qualitative Study: Burden of Menopause-Associated Vasomotor Symptoms (VMS) and Validation of PROMIS Sleep Disturbance and Sleep-Related Impairment Measures for Assessment of VMS Impact on Sleep**

Marci English, Boyka Stoykova, Christina Slota,
Lynda Doward, Emad Siddiqui, Rebecca Crawford, Dana DiBenedetti

**Contents**

**Online Resource 1.** Description of Semi-Structured Interview Guide

**Online Resource 2.** Additional Representative Quotations

**Online Resource 1**

**Description of Semi-Structured Interview Guide**

The semistructured Interview Guide was developed by RTI Health Solutions (RTI-HS; Research Triangle Park, NC) in conjunction with Astellas to ensure that topics relevant to the study objectives (menopausal symptoms, impact of vasomotor symptoms [VMS], and Patient-Reported Outcomes Measurement Information System [PROMIS] measure review) were addressed in each interview and to allow data to be collected in a consistent and systematic manner. It was constructed in a way that encouraged a conversational tone and allowed for spontaneity of responses.

The Interview Guide directed interviewers to open the conversation with a brief reminder to participants of the purpose and format of the interview (as previously detailed in the Informed Consent) and to instruct them to “think aloud” in providing their answers. For the concept elicitation portion of the interview, the Interview Guide included questions about women’s current and initial menopause symptoms and their specific experiences with hot flashes and night sweats. Questions were included about the impact of menopausal symptoms in general and on specific aspects of women’s lives (sleep, social life, work, memory/concentration, mood, physical health, relationships, and self-care) and about which of these impacts was most bothersome.

For the cognitive debriefing portion of the interviews, the Interview Guide specified that the participants should first complete the PROMIS measures on their own. The Guide included specific probing questions designed to elicit their overall thoughts about each PROMIS measure, as well as specific thoughts about how well they could understand and answer the questions, the relevance of the items to their experiences with VMS, the comprehensiveness of the questionnaires, and the use of the 7-day recall period in answering the questions. The Interview Guide concluded with a question asking the participant if she had anything else to add about her experience with hot flashes/night sweats and thanking her for her participation.

**Online Resource 2**

# Additional Representative Quotations

|  | **US Participants** | **EU Participants** |
| --- | --- | --- |
| **VMS description** | “… like a wave of heat. And all of a sudden I’m just boiling … it kind of comes and…like a rollercoaster…it goes down again” –Chicago-3  “… as if something, a chemical is coming out of you and burning your skin … I get almost like you’re being strangled, like a pressure in your head” –Chicago-5  “Sometimes it’ll be like a downpour” –Chicago-7 | “… feeling of growing warmth, almost like an anxiety swell, something that rises and becomes more intense. I become all red … I get drenched in sweat, I have to stop whatever I am doing to get a hold of myself” –France-1  “I’m dead hot, I can’t get my breath and you think I am going to faint, and then all of a sudden it just goes off” –UK-7 |
| **Work impacts** | “I’ll be on the phone with a customer and I’m having a hot flash … I have to put them on hold.” –Dallas-4  “I had a massive heat flash and I had to leave early. It was 3:00. I was like ‘I’m done.’ I’m tired. I’m cranky. I’m hot.” –Dallas-3 | “I have a fan on my desk at work and I think that singles me out a bit as the older lady on the team. I feel a little bit stigmatized in that way.” –UK-5  “The worst thing is that you can’t concentrate sometimes … when you go hot and you think, I’ve got to get out of here, I want to go home, I’ve had enough.” –UK-7 |
| **Mood impacts** | “My oldest [son] says, ‘Oh, get away from her; right now she’s mean.’” –Dallas-9  The mood swings…it’s kind of everybody don’t understand what’s going on. So they tend to get a little offended. I call it standoffish…I don’t like being close” –Chicago-4 | “I just get to the point where I have chucked a book at the floor, I have got fed up with everything and done all the washing and just burst into tears” –UK-2  “If my family says something that I dislike, I will snap at them, I would pout, which is something that I did not do before” –France-6 |
| **Relationship impacts** | “I think it affects sometimes that intimacy if he’s touching me or attempting to … just cuddling, nurturing…it affects me because that’s when I don’t want to be touched. I’m too hot.” –Chicago-2 | “Definitely [avoid new relationships], because it’s the confidence thing with…the sweats” –UK-6  “ |
| **Social impact** | “It’s embarrassing…you feel like people are watching you. If I’m out with my friends on the weekend or with my fiancé, I’m like ‘let’s go back home’ because I’m not in the mood to do this and it’s only because I either have had a hot flash or I had one coming.” –Dallas-7 | “I hate going to hot climates … Years ago, I used to love going on the beach and I would lie in the sun for a long time, now I hate it.” –UK-1  “Sometimes you can feel really uncomfortable and irritable and you’re on a night out with friends and it can ruin that.” –UK-6  “You are eating at the restaurant with people you do not necessarily know, and then suddenly when you feel that it is coming, it is embarrassing … People are looking at you, talking to you, and then the sweat begins pearling on my face, I’m liquefying myself, and I don’t know what to do anymore.” –France-7 |
| **Focus/ concentration impact** | “I may be working and it’ll come up … I have to stop what I’m doing, go get some water, come back, breathe. And then start again so I can refocus because I might be sweating. If you’re really into what you’re doing, it could be kind of aggravating.” –Chicago-7 | “While you are thinking about that [hot flashes], you are not concentrating on what you are doing, so yes they would impact that kind of activity.” –UK-4  “I’m so tired [from the night sweats] that I was not able to concentrate anymore.” –France-4 |
| **Daily activities impact** | “I have to take a timeout with doing chores…It seems like the activity makes me sweat. Sweeping the floor, I stop, take a break, get some water, throw some water on my face.” –Chicago-4  “Like if I’m cooking and I got food on the stove…I have to hurry up and shut it off and go find a cool place. Because I’m just too uncomfortable to stand there.” –Chicago-6 | “When you’re really energic [sic], slim, you know [the] person that’s on the go all the time, painting, decorating, doing this, doing that and off to the gym and you can’t do it anymore, it’s just rubbish.”  –UK-6  “[The hot flashes] make me waste time. It’s annoying to me…it messes up the program.” –France-5 |
| **Physical health impacts** | “I think a couple of those times when I got so hot I felt dizzy. I even get nauseated.”  –Dallas-1  “I would be curious to have my blood pressure taken as I was having one. Because that’s what it feels like … your blood pressure is really, really elevated.” –Chicago-5 | “I got fatter, I gained some weight, that’s for sure. I used to practice sports, now I stopped. Always for the same reason … the hot flares are even more intense when I do a physical activity.” –France-1  “I have occasional headaches and they can be quite severe, more than they was before.”  –UK-8 |
| **Ideal VMS medication** | “[The ideal medication] would help me to sleep … because I think being sleep deprived touches so many other aspects of being able to function the next day.”  –Chicago-7  “Have them not be so long, going on for minutes and minutes. [The medication] may not be able to stop you [from] feeling them at all … but if it could decrease the level, that would probably be really good.” –Chicago-3 | “If [treatment] could take away some of the severity and they were still coming … then I think I could put up with a bit more.” –UK-6  “By reducing the intensity, you make it bearable. If you only reduce the frequency you can still wake up once during the night and it can be enough to prevent you from falling back to sleep … better to reduce the intensity, which would mean that it would reduce the impact of the hot flash and avoid being awakened by it.”  –France-4 |
